# Azithromycin for idiopathic acute exacerbation of idiopathic pulmonary fibrosis: a retrospective single-center study

**DOI:** 10.1186/s12890-017-0437-z

**Published:** 2017-06-19

**Authors:** Kodai Kawamura, Kazuya Ichikado, Yuko Yasuda, Keisuke Anan, Moritaka Suga

**Affiliations:** Division of Respiratory Medicine, Social Welfare Organization Saiseikai Imperial Gift Foundation, Inc., Saiseikai Kumamoto Hospital, Kumamoto, Kumamoto, 861-4193 Japan

**Keywords:** Interstitial pneumonia, Idiopathic pulmonary fibrosis, Acute exacerbation, Survival analysis, Azithromycin, Prognostic factors

## Abstract

**Background:**

Acute exacerbation (AE) of idiopathic pulmonary fibrosis (IPF) is a fatal condition without an established pharmaceutical treatment. Most patients are treated with high-dose corticosteroids and broad-spectrum antibiotics. Azithromycin is a macrolide with immunomodulatory activity and may be beneficial for treatment of acute lung injury. The objective of this study was to determine the effect of azithromycin on survival of patients with idiopathic AE of IPF.

**Methods:**

We evaluated 85 consecutive patients hospitalized in our department for idiopathic AE of IPF from April 2005 to August 2016. The initial 47 patients were treated with a fluoroquinolone-based regimen (control group), and the following 38 consecutive patients were treated with azithromycin (500 mg/day) for 5 days. Idiopathic AE of IPF was defined using the criteria established by the 2016 International Working Group.

**Results:**

Mortality in patients treated with azithromycin was significantly lower than in those treated with fluoroquinolones (azithromycin, 26% vs. control, 70%; *p* < 0.001). Multivariate analysis revealed that the two variables were independently correlated with 60-day mortality as determined by the Acute Physiology and Chronic Health Evaluation II score (*p* = 0.002) and azithromycin use (*p* < 0.001).

**Conclusion:**

Azithromycin may improve survival in patients with idiopathic AE of IPF.

## Background

Idiopathic pulmonary fibrosis (IPF) is a progressive and irreversible fibrotic lung disease with a variable disease course. Most patients with IPF have a relatively slow clinical course, but up to 15% of patients experience an acute exacerbation of IPF (AE-IPF) each year [1], defined as an acute worsening or development of dyspnea and new bilateral ground-glass abnormality and/or consolidation on high-resolution computed tomography (HRCT). A recent epidemiologic survey of Japanese patients with IPF showed that the most common cause of death was AE-IPF [2].

The outcome of AE-IPF is very poor. The reported 1-month mortality rate is approximately 60% [3], and the reported in-hospital mortality rate ranges from 50 to 60% [1, 4].

AE-IPF lacks an effective pharmaceutical treatment. Current guidelines recommend that most patients with AE-IPF should be treated with corticosteroids [5], but no controlled trials support this recommendation. The International Working Group recently proposed a revised definition of and diagnostic criteria for AE-IPF [6]. Previous diagnostic criteria [7] recommended strict exclusion of other causes of acute worsening of respiratory disease, but new criteria [6] have permitted physicians to include patients with triggered AE in addition to idiopathic AE-IPF.

In studies of the treatment and outcomes of AE-IPF, almost all patients received empirical antibiotics in addition to corticosteroids despite the lack of controlled trials showing a benefit of empirical treatment [3].

Azithromycin is a macrolide with immunomodulatory properties and anti-inflammatory effects. Previous reports have described the effectiveness of macrolides in patients with serious conditions, such as severe pneumonia [8] and acute lung injury [9]. Until 2011, erythromycin was the only macrolide that could be used by intravenous injection in Japan, but erythromycin has many side effects and drug interactions, so we did not routinely use intravenous　erythromycin in daily clinical practice. In cases of suspected AE-IPF, we had used quinolone based antibiotics. Intravenous azithromycin has been approved for clinical use since September 2011 in Japan. Azithromycin is safer and easier to use than erythromycin, and since the publication of Walkey’s report [9], we routinely use azithromycin for patient with acute respiratory failure from July 2012. We previously reported that intravenous azithromycin was associated with improved outcomes in patients with AE of chronic fibrosing interstitial pneumonia [10]. However, that report had two major limitations: the very small number of patients treated with azithromycin and the inclusion of patients with nonspecific interstitial pneumonia and chronic hypersensitivity pneumonia.

Therefore, our objective in the present study was to assess the effect of azithromycin on survival of patients hospitalized with AE-IPF in our hospital. Our hypothesis was that in patients with idiopathic AE-IPF diagnosed according to the recently established 2016 AE criteria [6], azithromycin is associated with a lower 60-day mortality rate. The secondary objective was identification of the prognostic factors for idiopathic AE-IPF.

## Methods

This single-center retrospective study was approved by the institutional review board of Saiseikai Kumamoto Hospital. Written informed consent was obtained from all patients or surrogates in accordance with the Declaration of Helsinki.

### Patients

We retrospectively reviewed the medical records of 160 consecutive patients with AE of fibrosing interstitial pneumonia who were admitted to Saiseikai Kumamoto Hospital from April 2005 to August 2016. All data had been collected prospectively as part of an ongoing AE-IPF registry. In total, 85 patients who met the criteria for idiopathic AE-IPF were included in this study. The following patients were excluded from the analysis: those treated with anticancer drugs or radiation, with hematologic disease, in the terminal stages of advanced cancer and who refused treatment, with underlying collagen-vascular disease, with occupational or environmental exposure, and with a history of AE.

### Diagnosis of IPF and AE-IPF

IPF was diagnosed by consensus criteria [5]. We also included patients confirmed to have the following two features before AE [patients with features of possible usual interstitial pneumonia (UIP) and traction bronchiectasis on HRCT and no surgical lung biopsy as previously described [11].

The diagnosis of idiopathic AE-IPF was made based on the International Working Group criteria [6]. To exclude infection, all patients underwent cultures of sputum, blood, and urine; *Streptococcus pneumonia* and *Legionella* urinary antigen tests; rapid influenza diagnostic testing; and a beta-D-glucan assay. Bronchoalveolar lavage and endotracheal aspiration were not routinely performed in our hospital because of severe respiratory failure. Cytomegalovirus antigenemia testing was carried out in patients who had received long-term high-dose prednisone and/or immunosuppressant treatment. Measurement of the serum B-type natriuretic peptide concentration and echocardiography were performed to assess whether the respiratory failure was cardiogenic.

### Intervention

Before June 2012, no patients with idiopathic AE-IPF were treated with macrolides; all were treated with fluoroquinolone-based antibiotics. Since July 2012, we have used intravenous azithromycin for patients with AE-IPF within 24 h of AE diagnosis. Thirty-eight patients in this study were treated with an azithromycin-based antibiotic regimen, and 47 were treated with a fluoroquinolone (historical control group). Azithromycin therapy (500 mg/day) was continued for 5 days if there was no reason to cease administration, such as side effects and arrhythmia; however, discontinuation of azithromycin was at the discretion of the attending physician.

AE-IPF was treated with high-dose corticosteroid pulse therapy (methylprednisolone at 1000 mg/day for 3 days) in all patients. The corticosteroid dose was tapered after pulse therapy (0.5–1.0 mg/kg/day). Patients received prophylaxis against *Pneumocystis* except patients with intolerability

Selection of treatment options (i.e., whether to use mechanical ventilation or repeat high-dose steroid pulse therapy) was at the discretion of the attending physician, but the combined use of a fluoroquinolone and azithromycin was restricted.

### Data collection

Demographics, clinical features, clinical data on admission, and HRCT data were collected from the medical records. The presence of cardiovascular events among patients treated with azithromycin during hospitalization was recorded.

### Statistical analysis

We summarized the patients’ baseline characteristics using percentages for categorical variables and medians and interquartile ranges for continuous variables; comparisons between groups were performed using the Mann-Whitney rank-sum test and Fisher’s exact test. The survival time in days is reported as the median [95% confidence interval (CI)] and was calculated from the time of admission until mortality of any cause during the study period. Patients were censored if they were alive at 60 days. Survival curves were plotted using the Kaplan-Meier method. Log-rank tests were used to compare differences in survival.

Univariate analyses by Cox proportional hazard models were used to assess the relationship between mortality and the following variables; age, sex, surgical lung biopsy, prednisolone use prior to admission, immunosuppressant use prior to admission, antifibrotic drug use prior to admission, anticoagulants use prior to admission, honeycombing on HRCT and/or confirmation of UIP by surgical lung biopsy or features of possible UIP with traction bronchiectasis on HRCT, HRCT pattern of AE (diffuse or multifocal) [12], PaO_2_/FiO_2_, Acute Physiology and Chronic Health Evaluation II (APACHE II) score, use of mechanical ventilation during hospitalization, azithromycin use, beta-lactam combination use (5 days or more), serum KL-6 concentration, serum lactate dehydrogenase concentration, serum surfactant protein D concentration, and serum C-reactive protein concentration at admission. Variables associated with the outcome with a *P*-value of <0.1 in the univariate analysis were entered the multivariate analysis. The multivariate analysis was performed using a Cox proportional hazard regression model with a backward-selection procedure.

We also estimated the association between mortality and azithromycin treatment using the Cox proportional-hazards regression model via inverse probability of treatment weighting using the propensity score. The weights were based on the probability of receiving azithromycin. The propensity score was calculated from the logistic regression model including following variables: age, sex, surgical lung biopsy, prednisolone use prior to admission, immunosuppressant use prior to admission, antifibrotic drug use prior to admission, anticoagulants use prior to admission, patients with honeycombing on HRCT and/or confirmation of UIP by surgical lung biopsy or features of possible UIP with traction bronchiectasis on HRCT, HRCT pattern of AE (diffuse or multifocal), PaO_2_/FiO_2_, APACHE II score, azithromycin use, serum KL-6 concentration, serum lactate dehydrogenase concentration, serum surfactant protein D concentration, and serum C-reactive protein concentration at admission.

All tests were two-sided and performed at a significance level of 0.05.

All statistical analyses were performed with EZR (Saitama Medical Center, Jichi Medical University, Saitama, Japan) [13], which is a graphical user interface for R Version 3.2.2 (The R Foundation for Statistical Computing, Vienna, Austria).

## Results

**Fig. 1:**
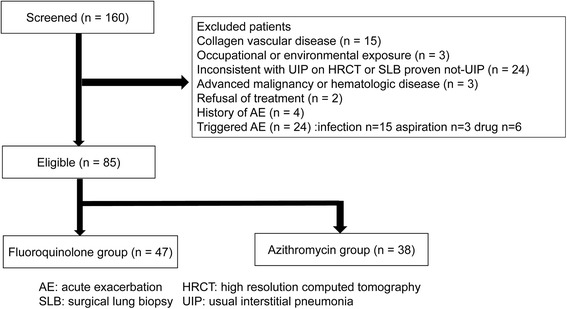
Patient flow diagram

A patient flow diagram is shown in Fig. 1. In total, 85 patients were enrolled. The baseline characteristics of patients with and without azithromycin treatment are summarized in Table 1. There were no significant differences in the patient characteristics between the azithromycin group and control group.

**Table 1:** Patients’ baseline characteristics

	All	Azithromycin	Fluoroquinolone	*p* value
	*n* = 85	*n* = 38	*n* = 47	
Age	76 (70–80)	75.5 (64–79)	76 (71–81.5)	0.65
Male sex	66 (78)	32 (84)	34 (72)	0.3
IPF diagnosis				
Honeycombing and/or surgical lung biopsy	78 (92)	35 (92)	43 (91)	1
Possible UIP with traction bronchiectasis	7 (8)	3 (8)	4 (9)	
Surgical lung biopsy	16 (19)	5 (13)	11 (23)	0.274
HRCT pattern of AE				
Diffuse	77 (91)	32 (84)	45 (96)	0.132
Multifocal	8 (9)	6 (16)	2 (4)	
Corticosteroid use before admission	39 (46)	17 (45)	22 (47)	1
Immunosuppressant before admission	19 (22)	7 (18)	12 (26)	0.6
Antifibrotic drug before admission	15 (18)	10 (26)	5 (11)	0.086
Anticoagulant drug before admission	6 (7)	2 (5)	4 (9)	0.69
APACHE II score	16 (13–18)	15.5 (13–18)	16 (13–18)	0.546
PaO_2_/FiO_2_	165 (99–243)	159 (109–241)	167 (97–236)	0.933
Lactate dehydrogenase (IU/L)	340 (291–408)	323 (281–385)	355 (308–442)	0.079
C-reactive protein (mg/dl)	6.4 (3.0–11.1)	6.0 (2.9–9.0)	7.7 (3.2–12.6)	0.482
KL-6 (U/ml)	1280 (860–2110)	1085 (760–1703)	1380 (887–2430)	0.088
Surfactant protein D (ng/ml), *n* = 75	336 (222–515)	336 (248.5–567.5)	333 (211–494)	0.527

Data are presented as *n* (%) or median (interquartile range)

*Abbreviations*: *UIP* usual interstitial pneumonia, *HRCT* high-resolution computed tomography, *AE* acute exacerbation, *APACHE II* Acute Physiology and Chronic Health Evaluation II

The therapeutic interventions are summarized in Table 2. All patients were treated with high-dose corticosteroid pulse therapy. The median duration of azithromycin use after AE-IPF diagnosis was 5 days (IQR, 5-5 days); the mean duration was 4.8 ± 0.6 days. There was a significant difference in the use of invasive mechanical ventilation between the two groups [azithromycin, 0 (0%) vs. control, 6 (13%); *p* = 0.03]. High-flow nasal cannula tended to be more frequently performed in the azithromycin group than in the control group, but this difference was not statistically significant (*p* = 0.09). There was no significant difference in the use of non-invasive mechanical ventilation between the two groups (*p* = 0.11).

**Table 2:** Therapeutic interventions and outcomes and causes of death in the study cohort

	Azithromycin *n* = 38	Fluoroquinolone *n* = 47	*p* value
High-dose steroid pulse therapy	38 (100)	47 (100)	1
Beta-lactam combination:	6 (16)	5 (11)	0.5
PMX	0 (0)	2 (3.5)	1
Invasive mechanical ventilation	0 (0)	6 (13)^a^	0.03
Noninvasive mechanical ventilation	10 (26)	21 (45)	0.11
High-flow nasal cannula	3 (8)	0 (0)	0.09
DNI	19 (50)	32 (68)	0.12
60-day mortality	10 (26)	33 (70)	<0.001
Respiratory death	10/10 (100)	33/33 (100)	1

Data are presented as *n* (%)

*Abbreviations*: *PMX* polymyxin B-immobilized fiber column-direct hemoperfusion, *DNI* do not intubate

^a^Five patients underwent NIMV before IMV

**Fig. 2:**
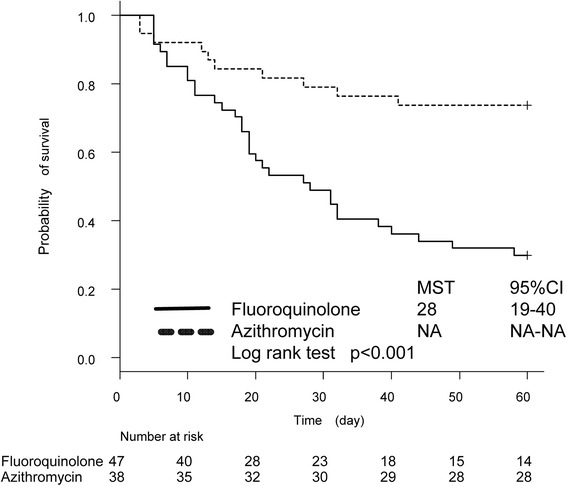
Kaplan–Meier survival curves for patients with idiopathic acute exacerbation of IPF treated with azithromycin or fluoroquinolone

The 60-day mortality rate was significantly different between the two groups (Table 2, Fig. 2). The mortality rate in patients treated with azithromycin was significantly lower than in those treated with a fluoroquinolone (azithromycin, 26% vs. control, 70%; *p* < 0.001; HR, 0.28; 95% CI, 0.14–0.57). The cause of mortality was respiratory failure in all of these patients.

The univariate analysis revealed six significant risk factors for 60-day mortality: the APACHE II score, PaO_2_/FiO_2_ ratio, serum lactate dehydrogenase level, serum C-reactive protein concentration, use of non-invasive mechanical ventilation, and use of azithromycin (Table 3). The multivariate analysis revealed that two variables were independently correlated with 60-day mortality: the APACHE II score (HR, 1.1; 95% CI, 1.01–1.19; *p* = 0.002) and azithromycin use (HR, 0.29; 95% CI, 0.14–0.60; *p* < 0.001) (Table 4). Inverse probability of treatment weighting with propensity score adjustment showed that azithromycin use was associated with lower mortality (HR, 0.28; 95% CI, 0.13–0.61; *p* = 0.001).

**Table 3:** Univariate analysis for 60-day mortality

	HR	95% CI	*p* value
Age	1.02	0.98–1.05	0.31
Male sex	0.74	0.38–1.45	0.38
IPF diagnosis			
Possible UIP with traction bronchiectasis	1	0.36–2.80	0.99
Surgical lung biopsy, Yes	1.08	0.52–2.26	0.83
HRCT pattern of AE			
Multifocal	0.41	0.10–1.71	0.22
Corticosteroid use before admission	0.75	0.41–1.38	0.38
Immunosuppressant before admission	1.19	0.60–2.36	0.62
Antifibrotic drug before admission	1.11	0.51–2.38	0.80
Anticoagulant drug before admission	1.40	0.50–3.91	0.52
APACHE II score	1.12	1.03–1.21	0.008
PaO_2_/FiO_2_	0.99	0.99–1	0.04
Lactate dehydrogenase (IU/L)	1	1.001–1.006	0.002
C-reactive protein (mg/dl)	1.05	1.0–1.1	0.04
KL-6 (U/ml)	1	0.99–1	0.49
Surfactant protein D (ng/ml), *n* = 75	1	0.99–1	0.23
Invasive mechanical ventilation	2.34	0.92–5.98–3.52	0.07
Noninvasive mechanical ventilation	1.85	1.01–3.37	0.047
High flow nasal cannula	0.53	0.07–3.89	0.54
Combination beta lactam	0.74	0.29–1.87	0.53
Azithromycin use	0.28	0.14–0.57	<0.001

*Abbreviations*: *IPF* idiopathic pulmonary fibrosis, *UIP* usual interstitial pneumonia, *HRCT* high-resolution computed tomography, *AE* acute exacerbation, *APACHE II* Acute Physiology and Chronic Health Evaluation II

**Table 4:** Factors independently associated with mortality after Cox regression

	HR	95% CI	*p*-value
APACHE II score	1.1	1.01–1.19	0.002
Azithromycin use	0.29	0.14–0.60	<0.001

*Abbreviations*: *APACHE II* Acute Physiology and Chronic Health Evaluation II

Serious cardiovascular events were not observed in the azithromycin group during hospitalization.

## Discussion

This single-center retrospective study of patients with idiopathic AE-IPF demonstrated that treatment with azithromycin within 24 h of diagnosis improves survival compared with treatment with fluoroquinolones.

The results in the present study are consistent with those in a previous report. We previously reported that intravenous azithromycin (500 mg/day for 5 days) was associated with improved outcomes in patients with AE of chronic fibrosing interstitial pneumonia without serious adverse events [10]. Our previous study that assessed the effectiveness of macrolides for treatment of AE of fibrosing interstitial pneumonia included not only patients with IPF but also those with nonspecific interstitial pneumonia and chronic hypersensitivity pneumonitis; AE of these conditions might be associated with a different mortality rate than AE of IPF. We included only patients with idiopathic AE-IPF in the present study; however, the effectiveness of azithromycin for AE was reproduced. A recent study using a large contemporary and comprehensive Japanese clinical database assessed the efficacy of combined treatment options including high-dose corticosteroids in patients with IPF with severe rapid progression of respiratory failure who required ventilator support. The study revealed that treatment with macrolides in combination with high-dose corticosteroids was significantly associated with a good prognosis [14]. This supports the findings of the present study.

Recent retrospective studies have revealed an in-hospital mortality rate of 50 to 58% in patients with AE-IPF [1, 4]. In the present study, the control group receiving fluoroquinolone-based treatment exhibited a similar survival rate. The 60-day mortality rate in patients treated with azithromycin was around 25%, and azithromycin treatment was a significant prognostic factor for survival in the Cox regression model. These findings suggest that treatment with azithromycin is effective for idiopathic AE-IPF.

This study focused on idiopathic AE-IPF. Previous diagnostic criteria for AE-IPF were limited to idiopathic cases [7]. However, newly introduced diagnostic criteria include patients with a trigger for AE [6]. Acute respiratory distress syndrome (ARDS) is a form of noncardiogenic pulmonary edema caused by alveolar injury secondary to an inflammatory process that can be both pulmonary and systemic in origin [15]. AE-IPF may be interpreted as ARDS that develops in patients with IPF. Like AE-IPF, ARDS has no established pharmacological treatment. A recent secondary analysis of multicenter-based randomized controlled trial data from the Acute Respiratory Distress Syndrome Network Lisofylline and Respiratory Management of Acute Lung Injury Trial showed that use of macrolides was associated with a lower 180-day mortality rate and shorter time to successful discontinuation of mechanical ventilation [9]. We validated the same effect in patients with septic ARDS [16]. These results suggest that macrolides (azithromycin) may be effective empirical antibiotics for patients with AE-IPF, which may be either idiopathic or triggered.

In a bleomycin-induced pulmonary fibrosis model, azithromycin reportedly reduced both fibrosis and restrictive lung function [17]. The authors of that study reported that besides the known effects of azithromycin on neutrophils (innate immunity), azithromycin also had a modulating effect on the TH1/TH2 fate as well as the TH17/iTreg lineage (adaptive immunity) [17], suggesting a potential role for azithromycin in the treatment of IPF. Cai et al. reported that alveolar macrophages in cryptogenic organizing pneumonia produces abundant proinflammatory cytokines and clarithromycin and AZM inhibit the production of aberrant proinflammatory cytokines by alveolar macrophages in a dose-dependent way [18]. Surgical lung biopsy in patients with AE-IPF generally identifies acute and organizing diffuse alveolar damage (DAD), with a predominance of organizing pneumonia [19, 20]. The hypothesis of a direct effect of macrolides on these organizing pneumonia areas or diffuse alveolar damage by reducing the priming of alveolar macrophage may be put forward.

The survival rate among patients who experience their first AE-IPF episode has improved; consequently, some patients experienced two or more AE episodes, which is very rare because historically, almost all patients with IPF died at the time of their first AE event. Pulmonologists must pay close attention to the care of patients with AE-IPF. Prevention of exacerbations is an essential strategy for improvement of both the IPF-associated mortality rate and quality of life. A recent randomized placebo-controlled trial showed that azithromycin significantly decreased the rate of exacerbations in patients with chronic obstructive pulmonary disease [21]. One small study showed that the use of azithromycin in patients with IPF was associated with a lower rate of admission for AE-IPF [22]. Further studies to assess the effectiveness of maintenance treatment with azithromycin are needed.

The strengths of our study are the inclusion of multiple potential confounding variables, the performance of sensitivity analyses with different methods of confounding adjustment using standard regression, and the propensity score-based inverse probability of treatment weighting.

There were some limitations to this study. First, this was a single-center, retrospective, observational study. Second, the study period was long and fluoroquinolone-based regimen was done before azithromycin-based regimen. Improvements in supportive care may have occurred over the 10-year study period that caused differences in outcomes between patients who were treated with a macrolide and those who were not. Head-to head comparison is needed to overcome this limitation. Third, baseline pulmonary function data prior to AE were not collected. Our hospital plays a central role in treating emergency patients in the surrounding area. Thus, there were many patients that were transferred to our hospital because of acute exacerbation of interstitial pneumonia from other hospital or had no diagnosis of interstitial pneumonia before AE-admission. Thus, there were many cases without pulmonary functional data prior to AE in our cohort. It would be important to investigate changes in FVC 6 months prior to AE-IPF in order to correct the predicting model per “disease activity”. This is essential in order to avoid survival considerations which did not take into account the dynamics of the disease. Forth, the sample size was small. We adjusted for overt biases by applying inverse probability of treatment weighting using propensity scores to resolve problems created by unequal chances of receiving treatment. However, we cannot rule out the possibility that unmeasured confounding factors might have affected our results. A large prospective study is needed to verify our results, but a randomized controlled trial may be difficult because of the low incidence of AE-IPF. Fifth, our result may not apply to all case of acute exacerbation. The new position paper of acute exacerbation of IPF do not require exclusion of triggered acute exacerbation, such as infection. In this study, we analyzed only patients with idiopathic acute exacerbation of IPF. Therefore, these results may not apply to patients experiencing AE-IPF secondary to infection. But as mention above, we reported the efficacy of azithromycin for septic-ARDS [16]. We speculate that our study could be extrapolated to infection induced acute exacerbation of IPF. Sixth, fluoroquinolone is an arbitrary control group. Reader should be kept in mind there might be possibility that fluoroquinolones have a potentially harmful effect on AE-IPF.

## Conclusion

In conclusion, our study results show that early administration of azithromycin may improve the survival of patients with idiopathic AE-IPF. Further studies are needed to verify our findings.

## Abbreviations

AE: Acute exacerbation
APACHE II: Acute Physiology and Chronic Health Evaluation II
HRCT: High-resolution computed tomography
IPF: Idiopathic pulmonary fibrosis
